# The Effects of Cold Atmospheric Pressure Plasma on Germination Parameters, Enzyme Activities and Induction of DNA Damage in Barley

**DOI:** 10.3390/ijms22062833

**Published:** 2021-03-11

**Authors:** Mária Peťková, Renáta Švubová, Stanislav Kyzek, Veronika Medvecká, Ľudmila Slováková, Andrea Ševčovičová, Eliška Gálová

**Affiliations:** 1 Department of Genetics, Faculty of Natural Sciences, Comenius University, Ilkovičova 6, Mlynská Dolina, 842 15 Bratislava, Slovakia; petkova21@uniba.sk (M.P.); andrea.sevcovicova@uniba.sk (A.Š.); eliska.galova@uniba.sk (E.G.); 2 Department of Plant Physiology, Faculty of Natural Sciences, Comenius University, Ilkovičova 6, Mlynská Dolina, 842 15 Bratislava, Slovakia; renata.svubova@uniba.sk (R.Š.); ludmila.slovakova@uniba.sk (Ľ.S.); 3 Department of Experimental Physics, Faculty of Mathematics, Physics and Informatics, Comenius University, Mlynská Dolina, 842 48 Bratislava, Slovakia; medvecka3@uniba.sk

**Keywords:** barley, cold atmospheric pressure plasma, DNA damage, enzyme activities, germination parameters, oxidative stress

## Abstract

Climate change, environmental pollution and pathogen resistance to available chemical agents are part of the problems that the food industry has to face in order to ensure healthy food for people and livestock. One of the promising solutions to these problems is the use of cold atmospheric pressure plasma (CAPP). Plasma is suitable for efficient surface decontamination of seeds and food products, germination enhancement and obtaining higher yields in agricultural production. However, the plasma effects vary due to plasma source, treatment conditions and seed type. In our study, we tried to find the proper conditions for treatment of barley grains by diffuse coplanar surface barrier discharge, in which positive effects of CAPP, such as enhanced germination or decontamination effects, would be maximized and harmful effects, such as oxidation and genotoxic potential, minimized. Besides germination parameters, we evaluated DNA damage and activities of various germination and antioxidant enzymes in barley seedlings. Plasma exposure resulted in changes in germination parameters and enzyme activities. Longer exposures had also genotoxic effects. As such, our findings indicate that appropriate plasma exposure conditions need to be carefully optimized in order to preserve germination, oxidation balance and genome stability, should CAPP be used in agricultural practice.

## 1. Introduction

Cold atmospheric pressure plasma (CAPP) is relatively widely used in agriculture and food production for the treatment of plants and seeds and also as a more environmentally friendly way for water desalination or decontamination [1,2,3] and for remediation [4,5]. Recent studies about CAPP effects are focused not just on water purification but also on plasma application on plants as a possible ecological solution for increasing food production. Climate change, environmental pollution and increasing pathogen resistance to available chemical agents represent some of the problems that the food industry has to face in order to ensure healthy food for people and livestock. One of the promising solutions to these problems is the use of CAPP. A number of available plasma sources and relatively simple treatment allow plasma to be applied to many agricultural products in postharvest or/and preharvest ways. Preharvest application primarily includes seed treatment in order to improve germination [6,7] and postharvest application includes treatment of final agricultural products such as fruits [8,9] or nuts [10,11,12,13], mainly for surface decontamination. In postharvest treatment, not only are decontamination effects of plasma important, but also physiological and morphological properties such as taste or color should be maintained.

There are many positive aspects of preharvest plasma treatment, e.g., surface decontamination [6,14], germination improvement [6,15], faster growth [13] and associated positive physiological and biochemical properties’ enhancement [16]. These effects were also observed on many different seeds treated with plasma generated by the same plasma source as in this study [16,17]. Active plasma compounds, including UV light or reactive oxygen and nitrogen species (RONS), are probably responsible for the positive effects of plasma. Through low levels of oxidative stress, plasma can eliminate pathogens presented on the surface of seeds [18] and it is impossible to develop resistance to plasma treatment as it causes serious damage in pathogen cells [19]. At the same time, plasma can change the intracellular concentration of RONS, which, as signal molecules, are able to modulate seed germination signal pathways [20,21].

Plasma treatment efficiency is variable and can change according to the plasma source, working gas, treated seed type and morphology of their surface [18], and for these reasons, it is almost impossible to determine general plasma treatment conditions for different seeds. The optimal plasma treatment time is specific for each seed species, as was observed in several studies [6,22,23], and probably depends on the type of seed, its size, surface hardness, embryo location, etc. However, Hthis is not the only problem with plasma treatment. Sometimes, the exposure time necessary for pathogen inactivation is too long and also causes damage to seeds, resulting in lower germination, as was shown in previous studies [24,25]. It was shown that longer exposure of seeds to plasma can cause serious damage to seed morphology that leads to reduced germination.

The aim of the present study was to identify treatment conditions for barley grains (*Hordeum vulgare* L.) by CAPP generated in different gases (ambient air, nitrogen and oxygen), causing no harmful effects and, at the same time, maintaining the positive ones (such as increased germination). Since the main active plasma components are RONS, our attention was focused on indirect oxidative stress markers, namely superoxide dismutase (SOD) and guaiacol peroxidase (G-POX), which detoxify oxidative stress products from cells. Determining the amount of DNA breaks and the presence of oxidized bases in DNA were also used as indirect evidence of oxidative stress induced by CAPP.

## 2. Results

### 2.1. Germination Parameters

**Figure 1:**
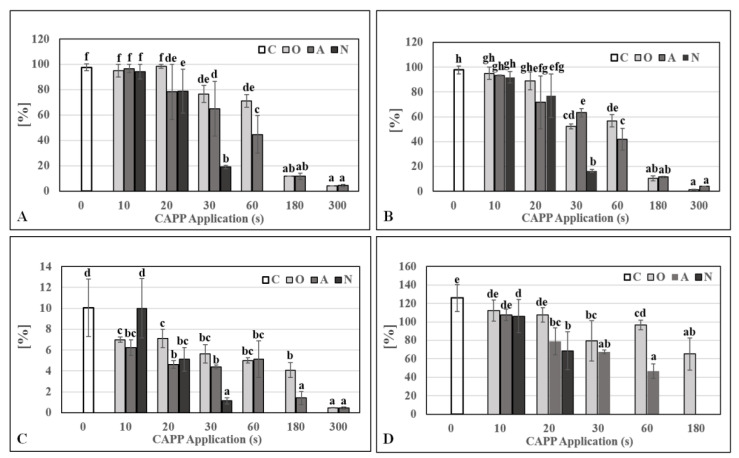
Germination (%) (**A**), Germination Potential (%) (**B**), Germination Index (%) (**C**) and Grain Vigor Index (%) (**D**) of barley grains after cold atmospheric pressure plasma (CAPP) treatment. Variants: C—control/untreated barley grains; O10–O300: barley grains treated with plasma generated in oxygen atmosphere for 10, 20, 30, 60, 180 or 300 s; A10–A300: barley grains treated with plasma generated in ambient air for 10, 20, 30, 60, 180 or 300 s; N10–N300 barley grains treated with plasma generated in nitrogen atmosphere for 10, 20, 30, 60, 180 or 300 s. Different letters indicate significant difference at *p*-value < 0.05, bars are means of ten experimental runs (one run represents 50 grains per variant, *n* = 500) ± SD according to Tukey’s HSD (Honestly Significant Difference) test. Indexes were calculated according to Abdul-Baki and Anderson [26].

In the case of cereal as barley, which has almost 100% germination (Figure 1) and retains it for many years, when stored correctly (in the dark, at a temperature of 8 °C), the use of cold atmospheric pressure plasma pre-sowing technology is important in the decontamination of grain surfaces from pathogenic microflora, faster recovery of metabolic activity in grains (lytic enzyme activation), faster growth and development of young seedlings and yield increase. Our results show that the CAPP treatment of barley grains had a negative effect on germination (%), germination potential (%), germination index (%) and grain vigor index (%) with increasing plasma application dose. The vigor of the grains did not decrease as dramatically as the germination potential and germination index. In the case of 10 s plasma application (for all working gases—O10, A10 and N10 variants) and in 20 s application (in the case of oxygen—O20 variant), the germination was comparable to the untreated control. In other variants (longer application time), we recorded a significant decrease in germination. For example, a more than sixfold reduction in germination rate was observed in O180 and A180 variants and a more than 10-fold reduction in germination rate in the O300 and A300 variants. CAPP generated in a nitrogen atmosphere completely inhibited the germination of barley grains at application dose 60 and more seconds (N60, N180 and N300 variants) (Figure 1).

**Figure 2:**
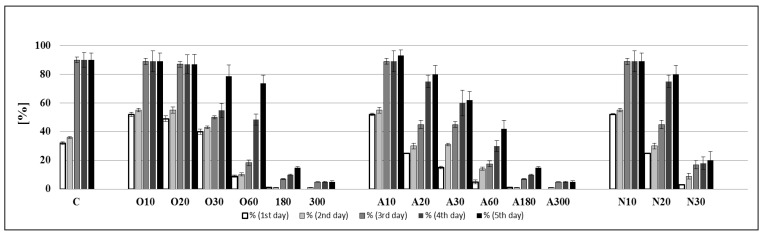
The dynamics of barley grain germination represented by calculated Percentage of Germination (%) on 1st, 2nd, 3rd, 4th and 5th day of cultivation. Variants: C—control/untreated barley grains; O10–O300: barley grains treated with plasma generated in an oxygen atmosphere for 10, 20, 30, 60, 180 or 300 s; A10–A300: barley grains treated with plasma generated in ambient air for 10, 20, 30, 60, 180 or 300 s; N10–N300 barley grains treated with plasma generated in a nitrogen atmosphere for 10, 20, 30, 60, 180 or 300 s. Bars are means of ten experimental runs (one run represents 50 grains per variant, *n* = 500) ± SD.

An interesting result was obtained in the monitoring of barley grains’ germination dynamics. Treatment with low doses of plasma (variants O10, O20, O30, A10 and N10) significantly accelerated germination on the first day of culture compared to the untreated control (Figure 2). This trend also continued on the second day of cultivation. However, on the third day, the number of germinated seeds in the variants treated with low doses of plasma and untreated control were comparable. At higher plasma application doses, germination was significantly delayed. The reduced vitality of barley grains and, thus, the reduced ability to germinate were also reflected in the production parameters (length and weight of roots and shoots) of 5-day-old seedlings (Table 1). The application of CAPP for 10 and 20 s for all working gases and 30 s for oxygen and ambient air had a slight stimulating effect on the weight and length of the roots and shoots. The action of CAPP generated in a nitrogen atmosphere for 30 s, in an oxygen atmosphere for 300 s and in ambient air for 180–300 s negatively affected the growth and development of roots and shoots. The seedlings were significantly deformed and stunted, so we have not presented data for these variants (N30, O300, A180 and A300) in Table 1 and they were not included in further experiments. The number of adventitious roots (in the case of variants that developed normally) did not change due to the CAPP application dose or working gas.

### 2.2. Enzyme Activities

**Figure 3:**
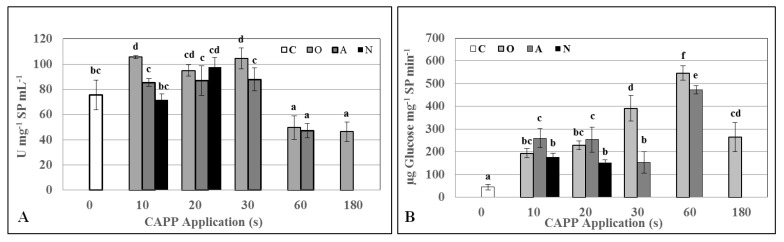
Activities of protease (**A**) and glucanase (**B**) in 3-day old barley seedlings after CAPP treatment. Variants: C—control/untreated barley grains; O10–O300: barley grains treated with plasma generated in oxygen atmosphere for 10, 20, 30, 60, 180 or 300 s; A10–A300: barley grains treated with plasma generated in ambient air for 10, 20, 30, 60, 180 or 300 s; N10–N300 barley grains treated with plasma generated in nitrogen atmosphere for 10, 20, 30, 60, 180 or 300 s. Different letters indicate significant difference at *p*-value < 0.05, bars are means of ten experimental runs (one run represents 50 seeds per variant; three 1.5-g mixed samples were analyzed per experimental run and each variant for protease and glucanase activities) ± SD according to Tukey’s HSD test.

**Figure 4:**
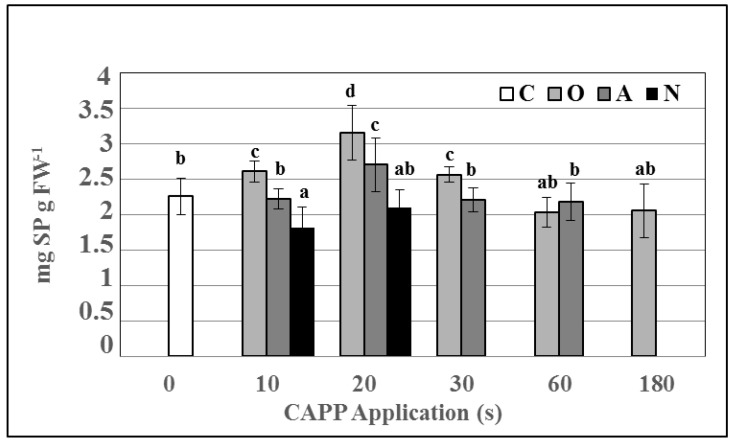
Total soluble protein concentration in 3-day-old barley seedlings after CAPP treatment. Variants: C—control/untreated barley grains; O10–O300: barley grains treated with plasma generated in an oxygen atmosphere for 10, 20, 30, 60, 180 or 300 s; A10–A300: barley grains treated with plasma generated in ambient air for 10, 20, 30, 60, 180 or 300 s; N10–N300 barley grains treated with plasma generated in a nitrogen atmosphere for 10, 20, 30, 60, 180 or 300 s. Different letters indicate significant difference at *p*-value < 0.05, bars are means of ten experimental runs (one run represents 50 seeds per variant; three 1.5-g mixed samples were analyzed per experimental run and each variant for soluble protein concentration) ± SD according to Tukey’s HSD test.

The activity of protease was positively affected after 10, 20 and 30 s treatment of plasma (for all working gases) and its activity decreased significantly with prolonged plasma exposure. Interestingly, glucanase activity was significantly higher in all plasma treated variants (compared to the untreated control), peaked at 60 s plasma treatment, and then decreased sharply (Figure 3). Using the Bradford method [27], a positive effect on the concentration of total soluble proteins in variants O10, O20, O30 and A20 was recorded (Figure 4).

**Figure 5:**
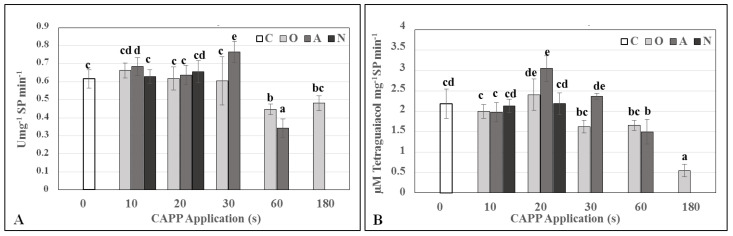
Activities of superoxide dismutase (SOD) (**A**) and guaiacol peroxidase (G-POX) (**B**) in 3-day-old barley seedlings after CAPP treatment. Variants: C—control/untreated barley grains; O10–O300: barley grains treated with plasma generated in oxygen atmosphere for 10, 20, 30, 60, 180 or 300 s; A10–A300: barley grains treated with plasma generated in ambient air for 10, 20, 30, 60, 180 or 300 s; N10–N300 barley grains treated with plasma generated in nitrogen atmosphere for 10, 20, 30, 60, 180 or 300 s. Different letters indicate significant difference at *p*-value < 0.05, bars are means of four experimental runs (one run represents 50 seeds per variant; three 1.5-g mixed samples were analyzed per experimental run and each variant) ± SD according to Tukey’s HSD test.

When monitoring the activity of antioxidant enzymes, lower plasma doses slightly stimulated superoxide dismutase activity (SOD), with statistically significant increases in SOD activity observed in variant A30. The activity of guaiacol peroxidase (G-POX) decreased slightly after 10 s treatment compared to the untreated control, peaked after 20 s treatment and subsequently began to decrease with increasing application dose (Figure 5).

### 2.3. Genotoxic Effects

**Figure 6:**
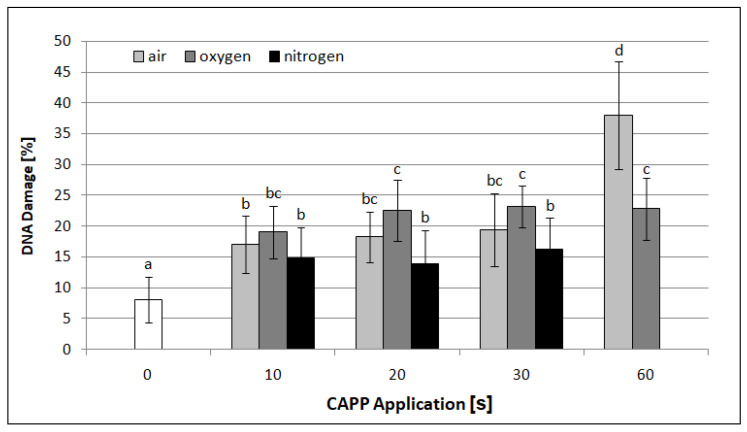
Graphical representation of the DNA damage effect of CAPP generated in ambient air, oxygen and nitrogen at exposure times of 10–60 s in barley leaves analyzed by comet assay. At least 100 nuclei were analyzed per slide. The data were analyzed using the statistical method LSD (Least Significant Difference) ANOVA and comparisons between the mean values were considered significant at *p* ≤ 0.05. Bars are means of three experimental runs ± SD.

The genotoxic potential of CAPP treatment on barley grains was estimated by standard and modified alkaline comet assays and constant field gel electrophoresis (CFGE) on 7-day-old seedlings. As shown in Figure 6, DNA damage was statistically significantly higher compared to control samples (8.08%) analyzed by comet assay at all exposure times. Moreover, the amount of DNA damage rose with increasing CAPP application. The highest rates of DNA damage were observed in samples treated by plasma generated in ambient air for 60 s (38%) and decreased at lower exposure times from 30 to 10 s (from 19.38% to 17.03%). DNA damage in samples treated with plasma generated in oxygen rose with increasing time from 19.08% (10 s) to 23.14% (30 s). The least DNA damage was observed in samples treated with plasma generated in nitrogen, where the values ranged from 13.91% (20 s) to 16.25% (30 s).

**Figure 7:**
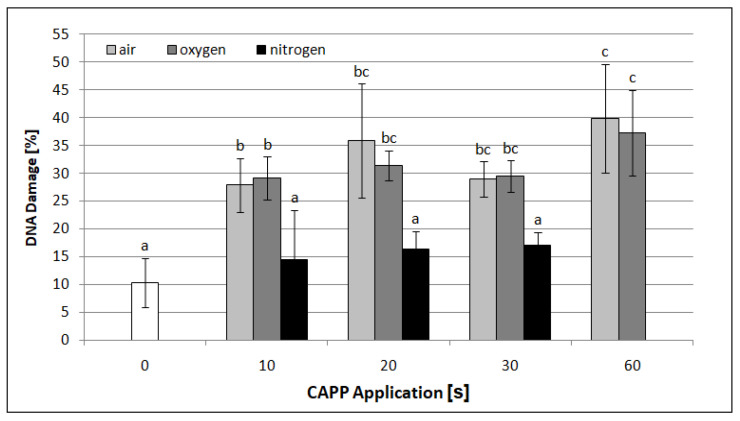
Graphical representation of the DNA damage effect of CAPP generated in ambient air, oxygen and nitrogen at exposure times of 10–60 s in barley leaves, analyzed by comet assay modified with formamidopyrimidine DNA glycosylase (Fpg). At least 200 nuclei were analyzed per slide. The data were analyzed using the statistical method LSD ANOVA and comparisons between the mean values were considered significant at *p* ≤ 0.05. Bars are means of three experimental runs ± SD.

The alkaline comet assay is the method used for the detection of DNA single- and double-strand breaks. Moreover, modification of this method using the formamidopyrimidine DNA glycosylase (Fpg) enzyme also enables the detection of oxidative damage of purines. In our study (Figure 7), we did not observe a statistically significant increase in DNA breaks and oxidation damage in samples treated with plasma generated in nitrogen compared to the negative control (10.3%). However, the rate of these types of DNA damage increased similarly in samples treated with plasma generated in ambient air and oxygen at all exposure times. A comparison of Figure 6 and Figure 7 shows that the values detected by comet assay modified with Fpg enzyme were higher than those by standard comet assay in all samples treated with plasma generated in ambient air or oxygen. These results suggest that treatment with these types of plasma resulted in purine oxidation. However, DNA damage analyzed by modified and standard comet assays did not reveal evident differences in samples treated with plasma generated in nitrogen, showing that this plasma type did not cause purine oxidation.

**Figure 8:**
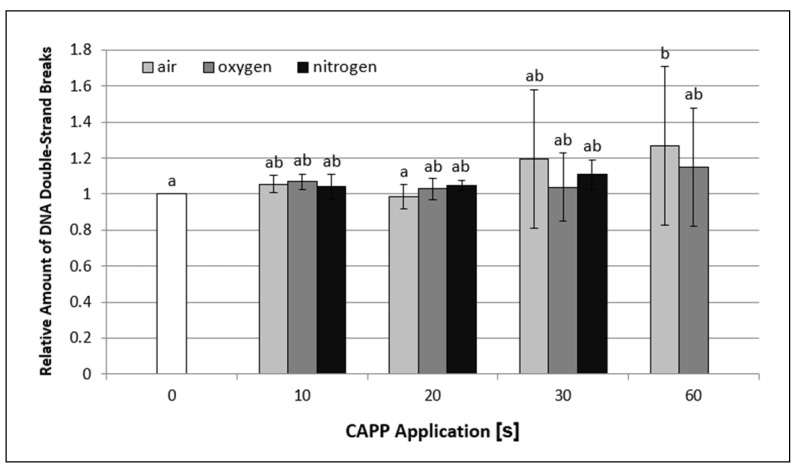
Graphical representation of the CAPP generated in ambient air, oxygen and nitrogen at exposure times of 10–60 s on the degree of double-strand breaks in barley leaves’ DNA, analyzed by constant field gel electrophoresis (CFGE). The data were analyzed using the statistical method LSD ANOVA and comparisons between the mean values were considered significant at *p* ≤ 0.05. Bars are means of three experimental runs ± SD.

As by comet assay, it is possible to detect both, single- and double-strand breaks, the CFGE method was used for double-strand break evaluation specifically. As shown in Figure 8, the relative amount of DNA double-strand breaks increased only at longer exposure times. The highest and only statistically significant increase compared to control samples was observed in 60-s exposure to plasma generated in ambient air (1.27-fold). These results suggest that CAPP treatment does not induce DNA double-strand break formation and that DNA damage observed by standard comet assay was mainly represented by single-strand breaks.

## 3. Discussion

It has been shown that plasma exposure affects seed germination differently depending on various factors including the type of the plasma source, working gas and exposure time [28,29], plant species [30] or even seed/grain cultivation conditions [31]. For example, while wheat grain germination was accelerated by cold plasma discharge treatment, both germination rate and germination speed of oat grains were unaffected by different time exposures to plasma [30]. Similarly, in our study, germination of barley grains (*Hordeum vulgare* L. cv. Maltz) was significantly accelerated after treatment with low doses of cold atmospheric pressure plasma (up to 30 s) on the initial days of cultivation compared to the untreated control. Park et al. [32] state that surface dielectric barrier discharge did not show a remarkable difference in the early germination beginnings of two barley cultivars (Saechal—waxy hull-less barley; Saessal—non-waxy hull-less barley cultivar developed in Korea). After artificial infecting of barley grains with pathogenic fungi of the genus *Fusarium* and subsequent plasma treatment with low-pressure Ar/N_2_-O_2_ surface-wave microwave discharges for 3 min, Szöke et al. [33] noted an increase in the germination of infected seeds above 80%. However, the germination and vigor of non-infected barley grains were not significantly affected after plasma treatment. Positive effects of non-thermal plasma application on seed germination (after optimal application doses) have been observed in a great variety of plant species, including monocots,—e.g., maize [34], wheat [30,35] and rice [36]—and dicot plants—e.g., *Chenopodium album* [37], pea [16,38], soybean [39,40] and tomato [31]. As in our study, the negative effect of high application rates of non-thermal plasma, and in particular, the negative effect of nitrogen as a working gas, has recently been described, e.g., for peas and soybeans [38,40,41,42]. The sensitivity of barley grains to prolonged plasma doses, which represent a highly reactive environment [42], can be explained by the thin coating of the grains. In addition, it was found that in contrast to its wild relatives, cultivated barley does not accumulate substantial amounts of proanthocyanidins (PAs) [43], which first condense into tannins and then oxidize to brown pigmentation seen in the mature seed of many species [44], and PAs are closely related to stress responses [45]. Several authors agree that the application of plasma can partially disrupt/modify the layers of seed/grain coatings, and thus, reactive particles can penetrate deeper and directly interact with the storage tissues or directly with the embryo [16,32,38,46].

We found that low doses of CAPP generated in various working gases slightly stimulate the activity of antioxidant enzymes (SOD and G-POX) involved in the detoxification of RONS. This is very important in managing oxidative stress. Moreover, our data show that it can also alleviate waterlogging stress, which is a major abiotic stress for barley [47]. Restoration of the metabolic activity of dormant seeds/grains is another important part of the germination story. In the case of cereals, the endosperm is an embryonic tissue highly specialized for nutrient storage, containing organelles (protein bodies and protein storage vacuoles) for the accumulation of storage proteins and sugars. Fully developed cereal endosperm represents approximately 75% of the grain weight and consists of four types of cell layers: aleurone, starchy endosperm, transfer cells and the cells of the embryo-surrounding region [48]. The starchy endosperm is characterized as a storage site, accumulating starch and seed storage proteins. The aleurone layers play essential roles during seed germination, where during imbibition, lytic enzymes [49] are activated by the action of gibberellic acid [50,51]. Many authors agree that the germination acceleration after plasma treatment is due to the faster activation of lytic enzymes [32,38,39,40,52]. Our results correlate with these findings and we confirmed that low plasma doses stimulate both protease and glucanase activities in barley grains. This resulted in a slight increase in the concentration of total soluble protein and better production parameters of young barley seedlings. Los et al. [53] reported improvement of production parameters (length of roots and shoots) of 7-day-old wheat seedlings after 30 and 60 s treatment with cold plasma. Park et al. [32] observed faster growth of plasma-treated barley hypocotyls (at least 15% or maximum 110%) than non-treated samples.

As we mentioned before, plasma decontamination effects are probably caused by RONS, which can attack nearby cells and oxidize macromolecules, even DNA. It is the reason why we focused also on the genotoxic potential of plasma using three methods for oxidized bases, DNA single- and double-strand breaks detection. According to our results, we suggest that the least harmful working gas for barley grains treatment is nitrogen, where the number of primary DNA damages was the lowest one from all monitored working gases. However, these results differ from other studies where the same plasma source, diffuse coplanar surface barrier discharge (DCSBD), for various seeds’ treatment was used. In the study of Švubová et al. [40], plasma generated from ambient air was the most suitable for soybean treatment. The number of DNA breaks in soybean seedlings after 30 s treatment with plasma generated in ambient air was on the same level as in control samples. Similar results were observed also in study of Tomeková et al. [42], where 60 s treatment with plasma generated in ambient air resulted only in a slight (1.6-fold) increase in DNA damage in pea seedlings. However, pea seed treatment with plasma generated from nitrogen caused approximately a fourfold higher level of DNA damage. Švubová et al. [38] observed the same results in single-strand break formation on pea seedlings after plasma treatment as in the previously mentioned studies. Similar to our study, the amount of double-strand breaks was comparable to the level of plasma-untreated samples. Indirect plasma effects on barley plants through plasma-activated water were observed by Ndiffo-Yemeli et al. [54] and, in comparison to our results, a significant increase in DNA damage was not observed. Differences in the results of our and other studies could be caused by different plasma treatment conditions, models organisms and their responses to plasma treatment. Single-strand break formation was observed also on the plasmid DNA treated by CAPP in many studies [55,56,57]. In the study by O’Connell et al. [55], CAPP generated from helium with oxygen addition primarily caused single-strand breaks in the plasmid DNA; however, with increasing treatment time, double-strand break formation occurred too. Moreover, with increasing concentration of atomic oxygen in plasma, the amount of double-strand breaks increased. This suggests that mainly atomic oxygen generated by plasma may be responsible for DNA break formation. Similar results were observed by Alkawareek et al. [56] on plasmid DNA treated by CAPP generated from helium with oxygen addition. The number of double-strand breaks in plasmid DNA was higher with increasing oxygen concentration in plasma. These studies suggest that DNA damage depends on the working gas. Reactive species present in the plasma generated in different working gases vary from each other in the stability and affinity to DNA [58]. The differences in our results and those of studies with plasmid treatment could be caused due to the fact that plasmid DNA is bare and unprotected. However, the DNA of plant cells interacts with proteins and is protected by a nuclear membrane, plasma membrane and cell wall.

## 4. Materials and Methods

### 4.1. Plant Material

The dried barley (*Hordeum vulgare* L. cv. Maltz) grains used in the experiments were obtained from the Central Control and Testing Institute in Agriculture in Bratislava, Slovakia. The seeds were stored at 8 °C in the dark.

### 4.2. Plasma Generation and Samples Treatment

Plasma is usually denoted as the fourth state of matter. Adding energy into a gas leads to ionization and formation of a chemically very active medium consisting of electrons, ions, excited atoms and molecules, metastable atoms and molecules and radiation [59]. For surface modification of materials, plasma is generated by electric discharges operating in a wide variety of electrodes’ geometry. The properties of plasma can be adjusted in terms of required processes by different working gases, modes of supply (frequency, voltage and input power), operating pressure, etc. [60,61].

The plasma treatment of barley samples was realized by a special type of atmospheric pressure discharge called diffuse coplanar surface barrier discharge (DCSBD) [62]. Unique design and optimized geometry allow the generation of low-temperature macroscopically uniform diffuse plasma in any working gas, even in electronegative, e.g., oxygen [63]. DCSBD consists of an alumina ceramic plate with two systems of parallel strip-like electrodes on the bottom side with alternating polarity of adjacent strips.

Plasma is generated in a thin layer on the surface of ceramics with active thickness in the range of 0.3–0.5 mm. The size of the discharge area (8 × 20 cm) enables the plasma treatment of a high amount of material. The generated plasma is characterized by high surface (1–3 W/cm^2^) and volume (~80 W/cm^3^) power density resulted in short exposure times [17].

**Figure 9:**
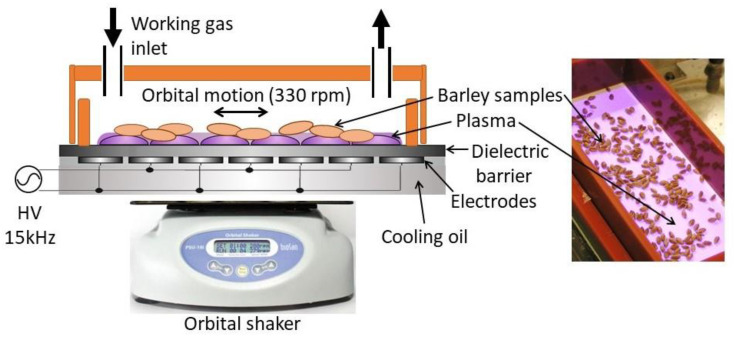
Scheme of the experimental apparatus with diffuse coplanar surface barrier discharge (DCSBD) and picture of barley samples’ exposure in plasma.

The experimental apparatus is shown in Figure 9. The DCSBD was placed on an orbital shaker and the homogeneity of samples’ treatment was ensured by the movement of samples at 330 rpm. The DCSBD was powered by a sinusoidal voltage of 20 kV (peak to peak) and a frequency of 15 kHz. The input power supplied to the DCSBD was 400 W and up to 50 barley seeds were placed in the DCSBD discharge area. The treatment was realized in a closed chamber in laboratory air and in oxygen or nitrogen in a flow regime with a flow rate of 3 Lpm and was the same for each working gas. Exposure times were 10, 20, 30, 60, 180 and 300 s for each working gas.

### 4.3. Germination and Growth Conditions

Dry barley grains (50 for each variant) were sown on Petri dishes on sterile filter paper, watered and cultivated in dark conditions in an incubator at the temperature of 24 ± 2 °C for 5 days. During cultivation, the number of germinated grains was counted and, on the third day, material for biochemical analyses (enzyme activity assays) was collected. After 5 days, the length (cm) and weight (mg) of shoots and roots were measured and the number of adventitious roots of young seedlings was counted. These data were used to calculate Germination (%), Germination Potential (%), Germination Index (%) and Grain Vigor Index (%) according to Abdul-Baki and Anderson [26]. Changes in germination during 5-day-long cultivation were used to express the germination dynamics of barley grains.

### 4.4. Total Soluble Proteins Content

Samples (~1.5 g) were ground in liquid nitrogen with a mortar and pestle and suspended in 50 mM Na-Phosphate protein extraction buffer with 1 mM EDTA (pH 7.8). After 15 min centrifugation (12,000× *g*), the supernatant was used for the determination of protein concentration according to Bradford [27]. Total soluble protein content was calculated as the amount of total proteins per gram of fresh matter from the calibration curve. As the protein standard, we used bovine serum albumin (BSA). All chemicals were purchased from Sigma-Aldrich Co.

### 4.5. Protease and Glucanase Activity Assays

Changes in the proteolytic activity in 3-day-old barley seedlings were determined by incubating 150 µL of an extracted protein sample with 150 µL of 2% (*w*/*v*) BSA in 200 mM glycine-HCl buffer (pH 3.0) at 37 °C for 1 h. The reaction was stopped by the addition of 450 µL of 5% (*w*/*v*) trichloroacetic acid. Samples were incubated on ice for 10 min and centrifuged at 20,000× *g* for 10 min at 4 °C. The absorbance of the supernatant was measured at 280 nm by a Jenway 6705 UV/Vis spectrophotometer (Bibby Scientific Ltd., Essex, UK). One unit of proteolytic activity is defined as an increase of 0.001 in the absorbance at 280 nm per min [64]. The activity of β-1,3-glucanase was assayed by measuring the release rate of reducing sugar from laminarin as substrate according to the methodology of Somogyi [65] and Nelson [66]. Absorbance was measured by a Jenway 6705 UV/Vis spectrophotometer at 660 nm. Total enzyme activity was calculated from the calibration curve as a reduction in glucose min^−1^ mg^−1^ SP. As a standard, we used glucose. All chemicals were purchased from Sigma-Aldrich Co.

### 4.6. Assays of Guaiacol Peroxidase (POX, E.C.1.11.1.7) and Superoxide Dismutase (SOD, E.C.1.15.1.1) Activities

The activities of enzymes that detoxify H_2_O_2_ (POX, E.C.1.11.1.7) and ˙O^2^¯ (SOD, E.C.1.15.1.1) were tested. The activity of guaiacol peroxidase (G-POX) was established according to Frič and Fuchs [67], and the activity of superoxide dismutase (SOD) according to Beauchamp and Fridovich [68]. One unit of SOD activity is the amount of proteins required to inhibit 50% initial reduction in nitrotetrazolium blue chloride (NBT) under the light. G-POX activity is expressed in µM of tetraguaiacol min^−1^mg^−1^ by molar extinction coefficient of tetraguaiacol (26.6). All chemicals were purchased from Sigma-Aldrich Co.

### 4.7. Alkaline Comet Assay

The alkaline comet assay is a method used for measuring DNA damage in eukaryotic cells. Using the alkaline comet assay and its modifications, it is possible to detect different DNA defects such as single-strand breaks, double-strand breaks, cross-links, apyrimidine and apurine (AP) sites [69,70]. Our experiments were performed according to Gichner et al. [71] with a few modifications [41,72]. Briefly, two leaves of 7-day-old seedlings for each sample were cut using a razor blade, ensuring DNA release in 175 µL of 0.4 M Tris-HCl buffer solution (pH 7.5) (Sigma-Aldrich Co.) because of the mechanical disruption of the cell and nuclear walls. The slicing and releasing of the DNA were realized in the dark and on ice. After that, 100 µL of the DNA and buffer suspension was mixed with 100 µL of 1% low melting point agarose (Roth). The final solution was then placed on a slide covered with 1% normal melting point agarose (Roth) and then covered with a coverslip. The coverslips were removed after 5 min and 40 µL of formamidopyrimidine DNA glycosylase (Fpg, the concentration of 0.2 U) for oxidized purine detection was pipetted on half of each slide. After that, the slides were covered by coverslips and incubated for 30 min at 37 °C. After incubation, the coverslips were removed and the slides were placed in an electrophoretic chamber filled with a cold electrophoretic buffer solution containing 1 mM EDTA (Sigma-Aldrich Co.) and 300 mM NaOH (Centralchem) for 8 min. After that, electrophoresis was launched at 1.25 Vcm^−1^ for 15 min at 4 °C. Slides were then neutralized three times with 0.4 M Tris-HCl buffer solution (pH 7.5) and stained with fluorescent dye ethidium bromide (0.05 mM, 80 µL for each slide) (Serva) for 5 min. DNA damage was observed using an OLYMPUS BX 51 fluorescent microscope with a green excitation filter UMWIG3 under 400× magnification. Plasma-untreated seedlings were used as a negative control.

### 4.8. Constant Field Gel Electrophoresis

Constant field gel electrophoresis (CFGE) is a genotoxicological method based on the incorporation of isolated nucleoids into agarose plugs that subsequently undergo lysis and electrophoresis at a constant and low voltage. In this way, it is possible to evaluate the relative amount of double-strand breaks produced by the action of a potentially genotoxic agent [73]. Our experiments were performed according to Švubová et al. [38]. At first, two leaves of 7-day-old seedlings for each sample were cut using a razor blade in 175 µL of 0.4 M Tris-HCl buffer solution (pH 7.5) (Sigma-Aldrich Co.). This process was realized in the dark and on ice. After that, 45 µL of the DNA and buffer suspension was mixed with 85 µL of 0.8% low melting point agarose (Roth). Subsequently, 85 µL of the final solution was transferred into 100 µL sample holder and cooled at 4 °C for 1 h. Solid agarose plugs were placed into glass tubes filled with 750 µL of lysis solution (pH 8) containing 2% N-laurylsarcosine (AppliChem), 0.1% proteinase K (Sigma-Aldrich Co.) and 0.5 M EDTA (Sigma-Aldrich Co.). After that, tubes with agarose plugs were incubated at 4 °C for 30 min, and after that, at 37 °C for 18 h. The lysis solution was then removed and agarose plugs were washed four times with TE (Tris-EDTA) solution (pH 8) containing 10 mM Tris base and 1.25 mM EDTA (both Sigma-Aldrich Co.). Agarose plugs were then incubated in the same TE solution for 30 min and, afterward, were placed in wells of 0.8% agarose gel. After that, the gel was placed in an electrophoresis chamber filled with TBE (Tris-borate-EDTA) solution (pH 8.3) containing 45 mM Tris base, 1.25 mM EDTA and 45 mM boric acid (all three from Sigma-Aldrich Co.). Electrophoresis was launched at 20 V for 48 h and the gel was visualized by the GelCapture program (version 4.24) using a DNR Bio-Imaging Systems Ltd. transilluminator. After that, the DNA amount was quantified using the ImageJ program, and final values of the relative damage rates were obtained by the ratio between migrated and total DNA amounts in gel normalized to the negative control (plasma-untreated samples) [74].

### 4.9. Statistical Analyses

Data were analyzed using Statgraphics Centurion XV v. 15.2.05 (StatPoint, Inc., Warrenton, VA, USA) and Excel (Microsoft Office 2007). Treatment effects were analyzed by means of ANOVA single-step multiple comparisons of means using means of LSD tests or Tukey’s test and comparisons between the mean values were considered significant at *p* ≤ 0.05. All experimental data in this work are from at least three independent experiments.

## 5. Conclusions

Low plasma doses (up to 20 s for ambient air and nitrogen and up to 30 s for oxygen) have a positive effect on the germination dynamics of barley grains. This is probably due to significant stimulation of the activity of lytic enzymes that cleave the storage substances in the aleurone layers and in the starch endosperm. Slight stimulation of antioxidant enzyme activity improves the detoxification of emerging reactive oxygen species, leading to better production parameters and an overall improvement in the vitality of young barley seedlings. However, reactive oxygen species generated during plasma treatment could have negative effects on DNA molecules, as was proved in our study. Therefore, cold atmospheric pressure plasma application can represent a new green technology for the improvement of surface decontamination and germination of various seeds and grains, but its potentially harmful effects need to be further investigated.

## Figures and Tables

**Table 1:** Shoot and root length/weight and adventitious root number of 5-day-old barley seedlings after CAPP treatment.

CAPP Application (s)	Average Shoot Length (mm)	Average Shoot Weight (mg)	Average Root Length (mm)	Average Root Weight (mg)	Adventitious Root Number
C (0)	11.3 ± 0.43 d	81.9 ± 17.2 e	6.21 ± 0.96 e	69.5 ± 16.1 c	6.66 ± 0.59 d
O10	10.2 ± 1.11 b	81.1 ± 19.3 de	7.49 ± 1.33 f	82.7 ± 22.2 de	6.40 ± 0.70 abcd
O20	10.3 ± 1.91 bc	79.4 ± 23.1 cde	5.38 ± 1.13 bcd	93.0 ± 23.6 e	6.18 ± 0.60 abc
O30	11.3 ± 0.88 cd	97.5 ± 21.7 e	4.17 ± 1.11 a	97.0 ± 15.7 e	5.85 ± 0.69 a
O60	5.0 ± 1.05 a	63.2 ± 13.3 ab	4.61 ± 1.32 ab	58.8 ± 17.2 ab	6.47 ± 0.68 bcd
O180	4.9 ± 0.98 a	65.0 ± 14.3 abc	5.12 ± 1.29 bcd	48.8 ± 15.5 a	6.17 ± 0.85 ab
A10	11.3 ± 1.11 d	86.4 ± 22.6 ef	6.55 ± 0.84 ef	84.7 ± 24.7 de	6.60 ± 0.70 bcd
A20	11.0 ± 1.55 bcd	87.3 ± 22.3 ef	6.48 ± 0.77 e	68.5 ± 19.6 bc	6.90 ± 0.57 d
A30	10.9 ± 1.01 bcd	87.8 ± 22.2 ef	4.70 ± 0.78 abcd	75.0 ± 21.4 cd	6.75 ± 0.46 bcd
A60	5.3 ± 1.01 a	54.7 ± 17.0 a	4.78 ± 0.99 abc	54.2 ± 17.3 ab	6.67 ± 0.93 d
N10	11.5 ± 0.63 d	80.1 ± 18.6 de	5.51 ± 1.25 c	76.4 ± 18.5 cd	6.51 ± 0.76 bcd
N20	10.9 ± 1.64 bcd	72.5 ± 17.7 bcd	5.45 ± 0.59 cd	53.3 ± 17.6 a	6.65 ± 0.85 cd

Variants: C—control/untreated barley grains; O10—O300: barley grains treated with plasma generated in an oxygen atmosphere for 10, 20, 30, 60, 180 or 300 s; A10—A300: barley grains treated with plasma generated in ambient air for 10, 20, 30, 60, 180 or 300 s; N10—N300 barley grains treated with plasma generated in a nitrogen atmosphere for 10, 20, 30, 60, 180 or 300 s. Different letters indicate significant difference at *p*-value < 0.05, data represent means of ten experimental runs (one run represents 50 grains per variant, *n* = 500) ± SD according to Tukey’s HSD test.
